# Supplementary material for: Use of diuretics in shock: Temporal trends and clinical impacts in a propensity-matched cohort study
Source: PLoS One. 2020 Feb 13;15(2):e0228274. doi: 10.1371/journal.pone.0228274 (PMC7018137; doi:10.1371/journal.pone.0228274)

**S1 Fig.** Comparison between total urine output in 6 hours before and after time zero in A) control group, B) diuretic group.

**A)**


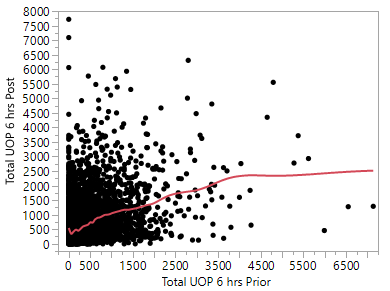


**B)**


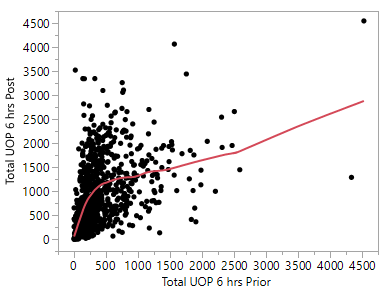

Supplement: S1 Fig — Comparison between total urine output in 6 hours before and after time zero in A) control group, B) diuretic group. (DOCX) [file pone.0228274.s001.docx]
